# Correlation between Oxidative Stress Markers and Periodontal Disease in Dogs

**DOI:** 10.3390/vetsci11030099

**Published:** 2024-02-22

**Authors:** Cosmin Petru Peștean, Hélène Pocquet, Daria Antonia Dumitraș, Andreea Georgiana Morohoschi, Laura Cristina Ștefănuț, Sanda Andrei

**Affiliations:** 1 Department of Surgical Techniques and Propaedeutics, Faculty of Veterinary Medicine, University of Agricultural Sciences and Veterinary Medicine of Cluj-Napoca, 400372 Cluj-Napoca, Romania; cosmin.pestean@usamvcluj.ro; 2 Department of Biochemistry, Faculty of Veterinary Medicine, University of Agricultural Sciences and Veterinary Medicine of Cluj-Napoca, 400372 Cluj-Napoca, Romania; helenepocquet@hotmail.com (H.P.); antonia.dumitras@usamvcluj.ro (D.A.D.); andreea-georgiana.morohoschi@student.usamvcluj.ro (A.G.M.); 3 Department of Animal Physiology, Faculty of Veterinary Medicine, University of Agricultural Sciences and Veterinary Medicine of Cluj-Napoca, 400372 Cluj-Napoca, Romania; cristina.stefanut@usamvcluj.ro

**Keywords:** dog, periodontal disease, MMP8, oxidative stress

## Abstract

**Simple Summary:**

In dogs, periodontal disease (PD) is a highly common condition whose prevalence rises significantly with age. The objective of this research was to assess the main indicators of oxidative stress detected in the saliva of dogs and to compare them to the degree of inflammation and tartar amount on their teeth and gums. The appearance of specific forms of periodontal disease has been confirmed by an increase in the saliva concentration of matrix metalloproteinase-8 (MMP8), a marker commonly used in humans in the diagnosis of these pathologies. Inflammatory processes in the oral cavity cause oxidative stress, demonstrated by the evaluation of different indicators in the saliva: an increase in the general antioxidant status, which was associated with an increase in superoxide dismutase (SOD) activity; an intensification of the lipid peroxidation processes; and an increase in the DNA oxidation processes.

**Abstract:**

In dogs, periodontal disease (PD) is a highly common condition whose prevalence rises significantly with age. Studies on rats with experimentally induced PD and human clinical trials have found an association between oxidative stress (OS) and PD, as has been observed in many other inflammatory disorders. The objective of this research was to assess the main indicators of oxidative stress detected in the saliva of dogs and to compare them to the degree of inflammation and tartar amount on their teeth and gums. By identifying these correlations, we intended to identify early-stage PD markers that would allow us to diagnose the condition in dogs without requiring for invasive or traumatic procedures. The antioxidant enzyme superoxide dismutase (SOD), total antioxidant capacity (TAC), malondialdehyde (MDA) and 8-hydroxyguanosine (8OHG), matrix metalloproteinase-8 (MMP8), and the quantity of total proteins are the markers that have been investigated in order to accomplish these objectives. The appearance of specific forms of periodontal disease has been confirmed by an increase in the saliva concentration of MMP8, a marker commonly used in the diagnosis of these pathologies in humans. The study was carried out on 22 dogs divided in a control group (C) and study groups (S), the second one being divided into three subgroups according to the severity of the gum inflammation and the presence or absence of tartar: S1—small accumulation of tartar, lack of infection-related signs; S2—moderate accumulation of tartar, gums swollen, red, and bled easily; S3—significant accumulation of tartar; gums bright red and bleeding; marked pain and halitosis. A correlation between the concentration of the tested parameters and the severity of the local appearance of the gum and teeth was noted. In comparison to the results of individuals from the control group, MMP8 reported increases of 1.2, 2, and 3.7 times, respectively, in the case of individuals from subgroups S1, S2, and S3. Oxidative stress is caused by inflammatory processes in the oral cavity. The presence of oxidative stress was demonstrated by the evaluation of different indicators in the saliva: an increase in the general antioxidant status, which was associated with an increase in SOD activity; intensification of the lipid peroxidation processes, as demonstrated by the accumulation of the MDA peroxidation product; and an increase in the DNA oxidation processes, as demonstrated by the accumulation of the oxidation product 8OHdG. In consequence, it was observed that there were up to 2-fold increases in protein levels, total antioxidant activity, SOD, and MDA, and up to 8.6-fold increases in the case of 8-OHdG when comparing dogs from the S3 group (significant tartar accumulation; bright red and bleeding gums; marked pain and halitosis) to those in the control group.

## 1. Introduction

Periodontal disease (PD), an inflammatory condition of the periodontium, which includes the gingiva, cementum, periodontal ligament, and alveolar bone that supports and surrounds the teeth, is one of the most common conditions in small animal practice [1,2]. However, there is limited information on the prevalence and severity of the disease with respect to different dog breeds. Furthermore, veterinarians frequently diagnose periodontal disease later in the disease’s progression [2,3]. Plaque is mainly caused by salivary glycoproteins adhering to teeth and forming a pellicle that becomes colonized by bacteria; the appearance of plaque is the earliest indication of the disease [2,4,5,6]. The mineralization of the plaque is known as tartar. It creates a suitable surface on which new plaque is able to develop [2,4,5,7,8].

Periodontal disease is classified into two types: gingivitis and periodontitis. Gingivitis is a treatable gingival inflammation, while periodontitis causes inflammation of all of the tissues that support teeth, leading to tissue damage and tooth attachment loss [2,4,7]. Gingivitis does not always progress to periodontitis, and therefore, it is possible that factors other than dental plaque (behavior, environment, inherent factors) promote PD [2,4,6,7,8]. The disease worsens over time since acute pain is rarely perceived in the early stages of PD. Pathological mandibular fractures may occur from alveolar bone loss, particularly among smaller breeds [7]. A first visual examination can be performed to evaluate the presence of gingivitis, plaque, tartar accumulation, root exposure, and missing or moveable teeth in order to identify periodontal disease in conscious dogs. To ascertain the kind and degree of PD, a dog examination under sedation is required. Alveolar bone loss can be evaluated using oral radiography [2].

The degree of periodontium destruction, periodontal pocket depth, gingival recession, and furcation exposure are used to measure the severity of periodontitis. In cases of gingival hyperplasia or inflammation, attention must be taken to avoid overestimating the loss of attachment [2,8,9]. The severity of PD is divided into four stages: (I) gingivitis; (II) early periodontitis, attachment loss < 25%; (III) moderate periodontitis, attachment loss 25–50%; and (IV) advanced periodontitis, with attachment loss > 50% [2,10]. It is important to know, however, that loss of attachment does not always equate to periodontitis, particularly in the absence of concomitant gingival inflammation. Furthermore, gingivitis is not always the initial sign of a subsequent loss of periodontal attachment [2,7,8,11,12,13,14].

It is suggested that PD is a site-specific disease. It appears that the maxillary teeth are more affected than the mandibular teeth [2,11,15,16]. Premolars and molars, particularly the first and fourth molars, are generally the teeth that are affected most frequently [2,11]. Additionally, it is possible that the teeth affected will vary depending on the breed of dog affected [2,14,16,17]. The first and most significant risk factor is thought to be inadequate dental care. Additionally, the specific type of bacteria involved influences the way the PD develops [2].

In dogs, a genetic predisposition has been proposed. Furthermore, research indicates that the prevalence and severity of the condition in dogs increase with age [2,11,14,15,16,17]. The toy (<6.5 kg) and the small (6.5–9 kg) and medium-small (9–15 kg) breeds of dogs tend to be more often diagnosed with PD. The medium large (15–30 kg), large (30–40 kg), and giant (>40 kg) breeds have a lower prevalence, except the Basset Hound and the Greyhound. The highest prevalence is recorded in Yorkshire terrier, then in Cocker spaniel, Jack Russell terrier, Border Collie, German shepherd, Labrador retriever and Staffordshire bull terrier breeds [2,18].

When examining the morphology of the jaw, tiny breeds’ teeth are proportionally larger than those of large breeds’, which increases the possibility of malocclusion and tooth crowding. Additionally, a link has been found between a higher frequency of PD and the thinness of the gingival and alveolar bone in very small dog breeds [2,19].

Several studies have identified an association between dog nutrition and PD. The risk of this condition is increased by a calcium deficiency. An elevated degree of PD is also connected with a lower body weight. According to certain research, dogs fed soft foods had higher levels of dental plaque and tartar, which increases the risk that the dogs might develop PD [2,20].

Maintaining clinically healthy gums is essential to avoiding plaque accumulation, tartar formation, and irritation [2]. Professional dental cleaning (under anesthesia) is the best way to remove the accumulated tartar, but this procedure should go with a well-built prevention strategy as the plaque is reformed only hours after the cleaning [2,7,21,22]. The best method of preventing plaque buildup is likely brushing the teeth at home, but it has to be performed according to certain recommendations to be successful [2,8,21]. According to Harvey et al. [7] and Quest [23], dental diet supplements, chews, gels, water additives, mouth rinses such as chlorhexidine, and chew toys can also be beneficial. When the condition has progressed to a late stage, the extraction of the tooth remains the best option [24].

The composition of the saliva varies according to the environment, the age, the feed, and the health status [25,26]. Salivary amylase is absent from dog saliva, but non-specific esterase, acid phosphatase, and pseudo-cholinesterase are present in large concentrations; immunoglobulin A is the most prevalent immunoglobulin, and glycosylated proteins are frequently detected [27]. Because of its composition, saliva is a complex biological fluid that is less difficult to collect than plasma and can be used to provide information about the health status of the animal.

Both the individual’s distinctive characteristics and the environment have an effect on the concentration of each of its components. One of these components, matrix metalloproteinase 8 (MMP-8), is an enzyme that is largely related to the initiation and development of PD. Since the relationship between this enzyme and the disease has been well established, MMP-8 is used in human dentistry as a PD biomarker. In humans, the increase in MMP-8 salivary concentration in the case of PD is stopped and even reversed after treatment. MMPs in general contribute to the induction of inflammation linked to tissue damage in PD and other inflammatory diseases. Other MMPs and increased oxidative stress levels are necessary for MMP-8 to become activated. Tissue inhibitors of matrix metalloproteinases (TIMPs) are responsible for controlling MMP levels. Defects in this regulation result in the extracellular matrix becoming damaged, the basement membrane, and alveolar bone deteriorating, all of which are factors that contribute to the development of periodontal disease [28,29].

Oxidative stress (OS) participates in normal aging as well as other pathologies. Oxidative stress interest grew following the association made between reactive oxygen species ROS (superoxide radical, hydrogen peroxide and hydroxyl radical, etc.) and their role in many diseases as well as their involvement in DNA damage and protein inhibition [30]. In order to protect themselves against the ROS, organisms developed numerous antioxidant systems [30,31]. Antioxidants are molecules that can delay or prevent oxidation of nucleic acids, proteins, lipids, or carbohydrates [32]. Different categories of antioxidant defense can be classified in three different groups: (1) enzymatic antioxidant (superoxide dismutase SOD, catalase CAT, glutathione peroxidase GPX, glutathione reductase and thioredoxin); (2) non-enzymatic antioxidants: water soluble (ascorbic acid, uric acid, bilirubin, glutathione, zinc, selenium, N-acetylcysteine, etc.) and lipid soluble (tocopherols, carotenoids, ubiquinol-10, melatonin); (3) the antioxidant proteins (albumin, haptoglobin, ferritin and ceruloplasmin) [30,31,33,34,35,36]. Biological fluids contain a variety of different antioxidants, and since many of them interact with one another, evaluating one antioxidant alone could compromise their efficacy and underestimate their true cumulative advantages. The measurement of biological fluids’ total antioxidant capacity (TAC) can help avoid this problem. TAC assesses the general state of antioxidants, both endogenous and those arising from exogenous ingestion, in the context of an increasing state of oxidative stress [37]. Cell membrane lipids, proteins, and DNA are all subject to oxidative damage, which is accelerated when ROS accumulate. Malondialdehyde (MDA), one of the byproducts of lipid peroxidation, is useful for measuring the effects of oxidative stress. Because 8-hydroxy-2’-deoxyguanosine (8-OHdG) is one of the most common modifications of nuclear and mitochondrial DNA caused by ROS, it is frequently utilized as a biomarker for oxidative stress.

According to the literature, previous investigations on oxidative stress and periodontal disease have already been conducted; however, none of those studies included dogs. This study aims to assess the main indicators of oxidative stress detected in the saliva of dogs varying in breed, age, and sex, and to establish a correlation between these markers and the degree of tartar and inflammation on their teeth and gums. By identifying these correlations, we intended to identify early-stage PD markers that would allow us to diagnose the condition in dogs without requiring for invasive or traumatic procedures. In order to achieve this, the following indicators have been investigated: total antioxidant capacity (TAC), malondialdehyde (MDA), 8-hydroxyguanosine (8OHG), antioxidant enzyme superoxide dismutase (SOD), metalloproteinase-8 (MMP8), and total protein.

## 2. Materials and Methods

### 2.1. Biological Material

A total of 22 dogs were included in the study. For each dog, we had the approval of the person legally in charge of the dog. The samples were taken at different places: private Clinique; Emergency Hospital, Faculty of Veterinary Medicine Cluj-Napoca; Animal welfare Association. A clinical examination was performed on each dog to evaluate its current state of health. Dogs having diseases that could be linked to oxidative stress were excluded from the study.

The animal study protocol was approved by the Ethics Committee of the University of Agricultural Sciences and Veterinary Medicine Cluj-Napoca, Romania (protocol no. 343/5 October 2022), according to the national law 43/2014 and EU Directive 2010/63/EU.

The following purebreds and mixes of breeds comprised the subject’s dogs: Icelandic sheepdog (1), Rottweiler (3), Belgian Tervuren (1), Australian shepherd (1), Border collie (1), French bulldog (1); and 14 mixed breeds dogs (mixed-breed × Romanian shepherd; mixed-breed × shepherd dog; mixed-breed Staff × Dogo argentino; mixed-breed Shepherd × Norwegian elkhound; mixed-breed × Shepherd dog; mixed-breed German shepherd × Belgian Malinois). Ten male and twelve females, representing the following age ranges, were included in the study: 2–3 years (6 dogs); 4–5 years (6 dogs); 6–7 years (4 dogs); 8–9 years (4 dogs); 14 years (2 dogs).

### 2.2. Clinical Examination

Conscious dogs underwent a clinical examination to visually evaluate the degree of gingivitis, plaque, and any evident indications gingival recession, root exposure, and missing or mobile teeth. The affected dogs with periodontal diseases were graded depending upon the severity of disease.

The veterinarians visually inspected the mouths of each dog. The American Veterinary Dental College (AVDC) scoring system has been modified and adapted for this purpose (Table 1). The affected dogs with periodontal diseases were graded depending upon the severity of disease.

Depending on the results of these examinations, the dogs were divided into two experimental groups: control group = clinically healthy dogs (C); and study groups = presence of tartar with or without associated inflammation observed on the teeth and gums.

### 2.3. Saliva Sampling Procedure

The samples were collected between 4 and 8 h after the last meal. In order to be able to collect saliva samples from dogs, we used cotton rolls with the following size: 4 cm width; 0.9 cm diameter. The sample procedure involved holding a cotton roll by one of the ends using a clamp or a needle holder, placing the cotton roll a few centimeters away from the dog’s nose for five seconds, and allowing the dog to smell it. If the dog begins to lick the cotton roll after sniffing it, we keep the cotton roll close to the mouth to facilitate as much easy licking as possible. If the dog does not lick the cotton after smelling it, it was put in its mouth to make contact with its oral cavity (tongue and palate) and vestibular cavity (space between cheeks and teeth). The dog was then let to bite the cotton roll multiple times. The cotton was placed immediately in a tube to be centrifuged. After 15 min of centrifuging the samples at 3500 rpm, the saliva samples were separated.

### 2.4. Biochemical Analyses of Saliva

- Determination of MMP-8 concentration

The salivary levels of MMP-8 were assessed in all the three phases of the experiment using the commercially available MMP-8 ELISA Kit, RayBio^®^, Norcross, GA, USA. The sample preparation was carried out in accordance with the kit’s instructions. The results were expressed as ng/mL saliva.

- Determination of total proteins

Protein quantification was performed according to the Bradford method. The measurements were performed using the SPECTROstar^®^ Nano—BMG Labtech, Ortenberg, Baden-Württemberg, Germany. The results were expressed as mg protein/mL saliva.

- Determination of Total antioxidants capacity (TAC)

TAC was assessed employing a commercially available colorimetric kit purchased from Elabscience Biotechnology Inc., Houston, TX, USA. TAC represents the system’s overall antioxidant capability across all antioxidant types. The antioxidants can reduce Fe^3+^ to Fe^2+^. The cations Fe^2+^ react with phenanthroline and form stable complexes. The TAC capacity was determined by measuring the absorbance of the complex at 520 nm. The measurements were performed using the SPECTROstar^®^ Nano—BMG Labtech, Ortenberg, Baden-Württemberg, Germany. The results were expressed as U/mL saliva.

- Determination of Total Superoxide Dismutase (T-SOD) activity

In order to analyze the activity of total SOD in saliva, a colorimetric assay kit (Total Superoxide Dismutase (T-SOD) Activity Assay Kit (Hydroxylamine Method) Elabscience Biotechnology Inc., Houston, TX, USA). The principle of the kit is as follows: xanthine oxidase (XO) can catalyze WST-1 and reacts with superoxide anions to generate a water-soluble formazan dye. SOD can catalyze the disproportionation of superoxide anions, so the reaction can be inhibited by SOD, and the activity of SOD is negatively correlated with the amount of formazan dye. The measurements were performed using the SPECTROstar^®^ Nano—BMG Labtech, Ortenberg, Baden-Württemberg, Germany. The results were expressed as U/mL saliva.

- Determination of 8-Hydroxydeoxyguanosine (8-OHdG)

In order to analyze the concentration of 8-OHdG in saliva, an in vitro enzyme-linked immunosorbent assay was used (Elabscience Biotechnology Inc., Houston, TX, USA). The results were expressed as ng/mL.

- Determination of malondialdehyde (MDA)

Determination of the MDA concentration in different tissues and fluids can reflect the level of lipid peroxidation in cells and reflect the level of cellular damage indirectly. MDA was measured using an assay kit from Elabscience Biotechnology Inc., Houston, TX, USA. Thiobarbituric acid (TBA) and the unsaturated aldehyde MDA react to produce an MDA-TBA complex, which can be measured spectrophotometrically. The sample preparation was carried out in accordance with the kit’s instructions. The measurements were performed using the SPECTROstar^®^ Nano-BMG Labtech, Ortenberg, Baden-Württemberg, Germany. The results were expressed as nmoles/mL saliva.

### 2.5. Statistical Analysis

The results are presented as means and standard deviation. All statistical analysis was performed using GraphPad Prism 8 (San Diego, CA, USA) statistics program and Epi Info 7 software (CDC, Atlanta, GA, USA). One-way and two-way ANOVA analysis of variance were performed, followed by Dunnett’s multiple comparisons post hoc tests to determine statistical significance.

## 3. Results

### 3.1. Clinical Examination and Classification of the Dogs

**Figure 1:**
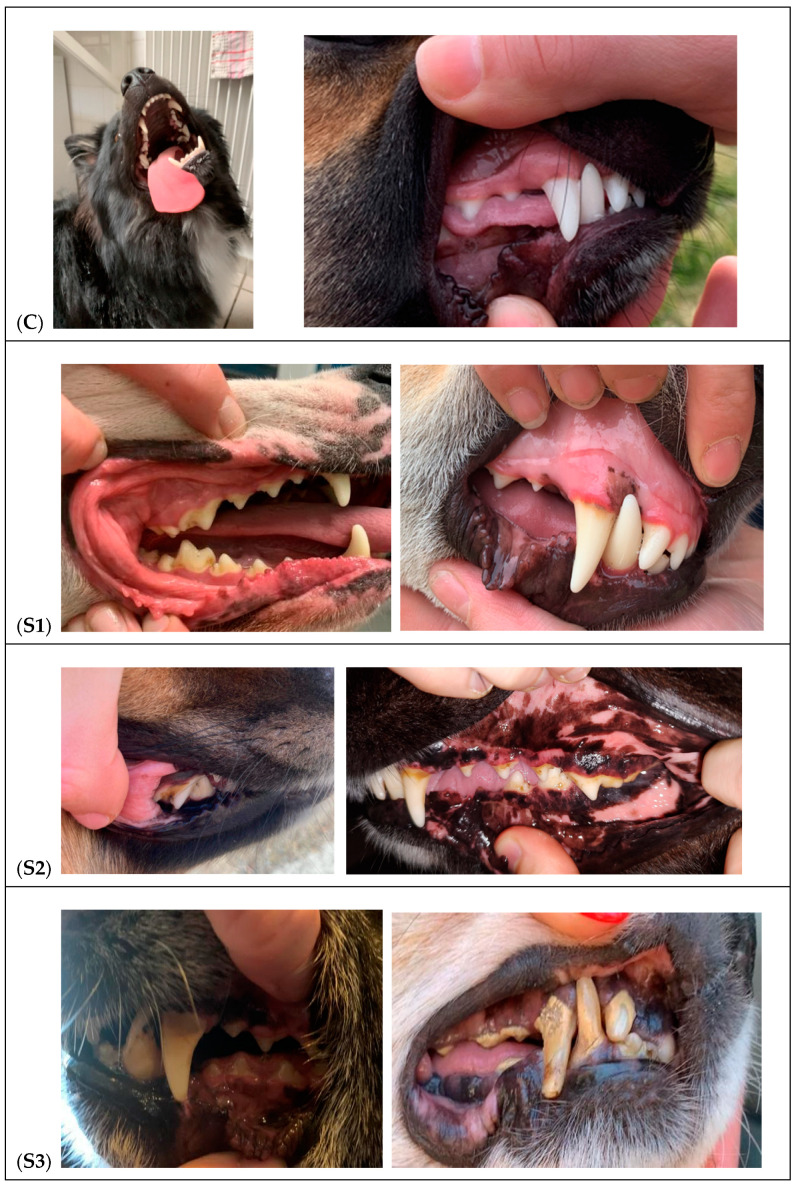
Clinical examination of the dog: (**C**) Control group teeth and gum; (**S1**) dogs with the lowest amount of tartar and possible light inflammation present on the gum; (**S2**) dogs with the low-medium amount of tartar and presence of a line of inflammation on the gum especially at the level of the canines; (**S3**) Dog of the study group with the high amount of tartar and continuous line of inflammation.

The objective of the clinical examination carried out on the dogs was to determine two important variables: the quantity and presence of tartar on the teeth, as well as the existence of gingival inflammation caused by the tartar. The dogs were divided into two groups, the control group and the study group, based on the presence or absence and intensity of each of these variables (Table 2 and Figure 1).

The dogs in the control group (6 dogs) had no accumulation of tartar and showed no signs of gum inflammation. A total of 16 dogs formed the study groups. Based on the quantity of tartar on their teeth and gum inflammation, the dogs in the study group were divided into 3 subgroups:

Group S1 (6 dogs): Soft plaque accumulates on the teeth’s surface. There was a small amount of tartar causes gingivitis by irritating the gums, lack of infection-related signs.

Group S2 (5 dogs): There was a moderate accumulation of tartar. The gums were painful, swollen, red, and bled easily. An odor was discernible in several cases.

Group S3 (5 dogs): Tartar and plaque formation was significant. The gums were bleeding bright red. Marked pain and halitosis was present.

Table 3 displays the subjects’ age and breed distribution within each group. The data indicates that the first signs and stages of periodontal disease are not correlated with the dogs’ ages. It was noted that the young subjects, who ranged in age from 2 to 5, were found in the S1 and S2 groups and the control group. On the other hand, the control group did not include subjects older than 5 years.

### 3.2. MMP8 and Total Proteins in the Dog’s Saliva

**Figure 2:**
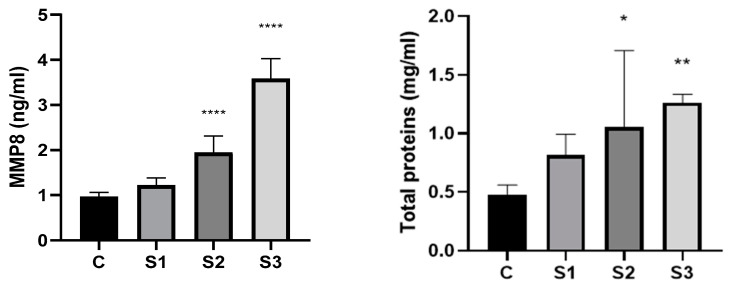
MMP8 and Total Protein concentration. C—control group; S1—study group 1; S2—study group 2; S3—study group 3. Values are expressed as mean ± standard deviation SD from three independent experiments. Statistical significance level * *p* < 0.05, ** *p* < 0.01, and **** *p* < 0.0001 (one-way ANOVA).

In our study, using the Elisa technique, we analyzed the level of MMP-8 in saliva collected from dogs, the results being presented in Figure 2.

Figure 2 represent the results of the analysis conducted to determine the protein concentration in the saliva samples.

### 3.3. Oxidative Stress Markers in Dog’s Saliva

**Figure 3:**
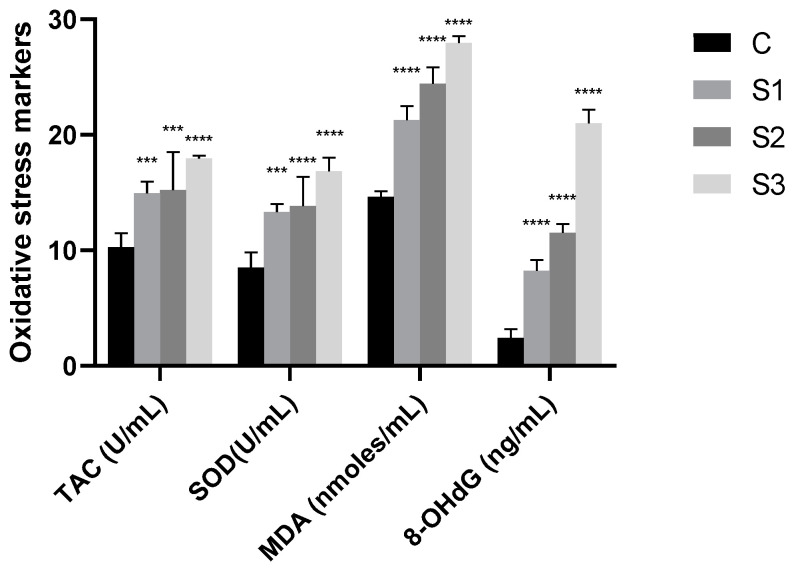
Oxidative stress biomarkers. C—control group; S1—study group 1; S2—study group 2; S3—study group 3. Values are expressed as mean ± standard deviation SD from three independent experiments. Statistical significance level *** *p* < 0.001 and **** *p* < 0.0001 (one-way ANOVA).

By accumulated oxidative species, oxidative stress is generated, which can further exacerbate oxidative degradation processes through a variety of pathways, including lipid peroxidation, DNA damage, oxidation of key enzymes, and protein damage. Table 4 displays the results of our investigation, which measured the antioxidant capacity (Total Antioxidant Capacity = TAC and SOD activity in U/mL), the levels of MDA (nmoles/mL) and 8OHdG (ng/mL) in saliva samples from the experimental and control groups. For a better visualization of the results for each biochemical parameter, a graphical representation of obtained data has been added (Figure 3).

## 4. Discussion

The most important salivary inflammatory biomarkers linked to oral disorders include matrix metalloproteinases (MMP-8 and MMP-9), tissue inhibitors of metalloproteinase, interleukins (IL-1, IL-6, and IL-8), and tumor necrosis factor (TNF-α) [38]. MMPs, often referred to as matrixins, are an important family of endopeptidases that contain zinc. Based on their structure and activity, these enzymes were divided into five sub-families: stromelizines, collagenases, gelatinases, membrane matrix metalloproteinases (MT-MMPs), and other MMPs [29]. The dogs in the study group had a significantly higher concentration of MMP8 than the control group, with double and triple values being recorded in the case of individuals from subgroups S2 and S3 (0.967 ± 0.090 vs. 1.949 ± 0.325; and 0.967 ± 0.090 vs. 3.579 ± 0.399) (*p* < 0.001). MMP-8 is a Collagenase2/Neutrophil Collagenase that shows substrate selectivity towards a variety of substrates, including Fibronectin, Aggrecan, Collagen I, II, and III, as well as ovostatin. Periodontal tissue turnover, anti-inflammatory action, and wound healing are the primary physiological roles; nevertheless, an increase in concentration has been linked to human cancer, rheumatoid arthritis, asthma, and periodontitis [29,38].

According to our research, higher levels of tartar and inflammation are closely related with elevated MMP-8 levels in saliva, which are indications of periodontal disease and inflammation. There are quite a few studies available on the levels of MMP-8 in canine saliva; the majority of investigations have concentrated on human and laboratory animal (rat) saliva. MMP-8 and MMP-9 have been linked to human periodontal disease, according to numerous research. In response to inflammatory conditions, neutrophils, smooth muscle cells, endothelial cells, and macrophages release neutrophil collagenase/MMP-8. In patients with periodontitis, there is a progressive loss of attachment correlated with salivary MMP-8 levels. Furthermore, compared to healthy controls, salivary levels of MMP-8 and IL-1 are substantially linked to severe periodontitis [38].

Between the experimental and control groups, there were statistically significant differences in the total protein concentration. By comparing the dogs in the study group to the control group, there is a significant increase in the concentration of proteins. Saliva samples from dogs with low tartar levels had an average protein concentration of 0.814 mg/mL, which was higher than the control’s 0.481 mg/mL. Furthermore, we observed an increase in protein content (1.057 mg/mL in the S2 group versus 0.814 mg/mL in the S1 group) along with the intensity of tartar development and gingival inflammatory processes. Regarding the total protein content in the saliva samples collected from the dogs in study groups S2 and S3, double concentrations were observed compared to those of individuals in the control group, the result being statistically significant as can be seen in Figure 3. Salivary protein content and, more precisely, profile are significantly impacted by the emergence of disorders specific to the oral cavity. Dental calculus is thought to be one of the primary factors in the development of periodontal disease in dogs, which leads to tooth loss. Saliva is a biological fluid that contains a wide range of proteins, with glycoprotein, enzymes, immunoglobulins, and numerous peptides being particularly well-represented. Because these proteins have distinct origins, the whole amount of saliva is composed of food particles, compounds produced by oral bacteria, and gingival crevicular fluid (which contains plasma proteins) [39]. Numerous factors impact the protein profile of dog saliva. Salivary flow rate can be increased and the proportion of proteins in saliva can be altered by adding stimulation while sampling the saliva [27]. The results reported by Pasha et al. [27] suggests that changes in salivary protein concentration are probably due to the breed of dog, which explains why the significant variances seen within the same group are normal. Furthermore, the breed of dog has a greater impact on the composition of salivary proteins than does the dog’s gender. Salivary protein content and, more precisely, profile are significantly impacted by the emergence of disorders specific to the oral cavity. Dental calculus is thought to be one of the primary factors in the development of periodontal disease in dogs, which leads to tooth loss.

Proteins in dog saliva are also more likely to be associated with antimicrobial drugs, which suggests that dog salivary proteins would have more antibacterial activity than human ones [26,40]. Saliva from dogs and humans has different proteome signatures. Research on the antimicrobial protein family in dog saliva revealed the presence of CRISP1, cathelicidin 1, and cathelicidin antimicrobial peptide. However, in healthy individuals, cathelicidins were not detected; however, CRISP3 appeared [25,26,41].

In the early stages of the PD disorder, we hypothesize that the accumulation of reactive oxygen species (ROS) triggers an antioxidant response. When the disease becomes more severe, the organism can no longer keep an equilibrium between the formation of ROS species and antioxidants and oxidative stress occurs. During the deterioration of periodontal tissue by polymorphonuclear leukocytes and macrophages, more ROS and free radicals are generated at the infection site. By preventing or postponing the oxidation of an oxidizable substrate, antioxidants provide local protection [33,37,42]. At the cellular level, there are numerous enzymatic and non-enzymatic antioxidants that function in a synergistic manner, making the assessment of the total oxidative and antioxidative capability important. An indicator of body fluids’ capacity for antioxidant activity is called Total Antioxidant Capacity (TAC) [43,44]. Data indicate that there is a correlation (Pearson coefficient r = 0.99) between the SOD activity and the total capacity measured in saliva samples. More specifically, as compared to the control group, the experimental groups exhibit higher levels of antioxidant activity. It was observed that there are no significant variations in total capacity and SOD activity between the saliva samples from dogs with low levels of tartar and no gum inflammation (S1) and those from dogs with high levels of tartar and inflammation (S2, S3). Studies focusing on human saliva have demonstrated that there is a significant difference in TAC levels between peripheral blood samples from patients with periodontitis (PD) and healthy subjects. These findings imply that systemic oxidative stress in human bodies is linked to chronic periodontitis. Salivary TAC values were often lower in PD patients compared to healthy people in 66.7% of published studies. It has been proposed that elevated salivary TAC levels may be an adaptive reaction to oxidative stress in PD [44]. In the study by Ahmadi-Motamayel et al. [45], salivary TAC of patients with dental caries and associated inflammation had higher than control group. There is a correlation between the results obtained in determining the proteins and the total antioxidant activity (TAC), demonstrated by the Pearson coefficient (r = 0.96). Salivary total antioxidant capacity (TAC) and salivary protein concentration have been shown to positively correlate in studies by Araujo et al. [46]. Salivary protein levels have been demonstrated to increase in persons with more severe dental caries. Matrix metalloproteinases (MMPs) are known to degrade the mineral and organic matrix in dentin lesions. High quantities of MMP-8 have been found in the saliva of dentin lesions patients. Salivary TAC has also been demonstrated to increase with the severity of carious lesions (either enamel or dentin).

The amount of tartar and the inflammation associated with the accumulation of tartar were directly correlated with MDA levels in our study. Other researchers have reported higher concentrations of compounds derived from lipid peroxidation in human saliva. For example, in the human saliva studies by Trivedi et al. [47], the MDA levels in saliva were significantly correlated with the periodontal clinical parameters in patients with varying degrees of periodontal disease. This suggests that the level of MDA increases as periodontal tissue destruction progresses.

8-hydroxydeoxyguanosine (8-OhdG), an oxidized nucleoside, is released through body fluids during DNA repair. Saliva from dogs with gingival injury and development of tartar showed higher levels of 8OhdG than saliva from dogs in clinical health (control). In contrast to other indicators examined in saliva, wherein dogs with varying levels of tartar (S1 versus S2 and S3) showed no significant variations, the increases in 8OhdG were directly associated with the severity of damage. Dog saliva from groups S2 and S3 demonstrates a statistically significant (*p* < 0.0001) increase in DNA oxidation product accumulation and oxidative degradation (Figure 3). The differences we observed in dogs and humans are comparable, despite our inability to compare the literature data on the accumulation of 8OHdG in dog saliva. However, we attempted to find this information through studies on human saliva. Sezer et al. [48] analyzed the levels of 8OHdG in saliva from patients who were clinically healthy as well as those who had chronic gingivitis and periodontitis. According to their research, patients with periodontitis had considerably greater salivary 8-OHdG levels than gingivitis patients or healthy controls. Çanakçi et al. [49] found similar results, suggesting that salivary 8-OHdG levels could indicate early oxidative mtDNA damage in injured gingival tissue in patients with periodontitis. Rheumatoid arthritis and inflammatory pulmonary disease are two more inflammatory disorders that might be compared to periodontal disease. ROS have a negative effect on tissues in these disorders as well [37,42].

Oxidative stress is a consequence of inflammation in periodontal disease and may be a major factor in the deterioration of the periodontium [37]. Furthermore, the critical impact that bacteria and other individual factors played in the disease’s tissue damage should not be ignored. For instance, in dogs, older age has a significant impact on the severity of the disease [37,50]. All humans as well as animals go through the natural process of aging, which is known to cause increasing amounts of DNA damage over time. As individuals age, their bodies produce more free radicals and less antioxidants overall than they did when they were younger. This leads to damage to DNA [37,51].

This study includes some limitations. First, the number of cases was small, thereby weakening the statistical analyses and increasing the chance of errors. We implemented exclusion criteria, leading to in a decrease in the total number of dogs included in the study. We only selected clinically healthy dogs who had been evaluated by veterinarians prior to samples collection to ensure that our study is free from any potential influence from other medical disorders that may be directly linked to oxidative stress. The inclusion of several dog breeds in our study represents an additional limitation. In order to avoid breed-related variations, more research could be carried out with dogs from kennels. Additionally, in kennels, additional factors that may cause variations in the saliva oxidative stress markers can be identified, such as the antioxidant content of the food or the dog’s diet. Nevertheless, more research with a larger number of samples as well as additional criteria must be conducted to further improve the clinical value of these salivary biomarkers in the surveillance and diagnosis of canine periodontal disease.

## 5. Conclusions

Dogs are more susceptible to acquiring periodontal disease due to tartar buildup in their teeth, which causes inflammation the gums. Tartar accumulation is directly correlated with the emergence of specific inflammatory processes. An increase in the concentration of MMP-8 in saliva, a marker commonly used in humans for the identification of various illnesses, has been utilized to validate the emergence of particular kinds of periodontal disease. Oxidative stress is brought on by oral cavity inflammatory processes. Multiple markers were evaluated in the saliva, and the results showed the presence of oxidative stress: increased SOD activity and total antioxidant status; increased lipid peroxidation processes as indicated by the accumulation of MDA peroxidation product; and increased DNA oxidation processes as indicated by the accumulation of oxidation product 8OhdG.

## Figures and Tables

**Table 1:** Rating system used for evaluation.

Stage	Description
O	No accumulation of tartar. Gums are normal.
I	Small accumulation of tartar. Gums are slightly inflamed.
II	Moderate accumulation of tartar. The gums are painful, swollen, red, and bleed easily.
III	Heavy tartar is present. Severe inflammation of the gums. Evidence of infection or tooth loss.

**Table 2:** Overview of the amount of tartar and inflammation level for each group.

Groups	Estimation of Tartar Amount	Estimation of the Degree of Inflammation
Control group C (*n* = 6)	–	–
Study group S1 (*n* = 6)	+	+
Study group S2 (*n* = 5)	+ +	+ +
Study group S3 (*n* = 5)	+ + +	+ + +

– No tartar and no inflammation; + reduce; + + moderate; + + + significant.

**Table 3:** A summary of the dog’s breed and age for each group.

Groups	Age	Breed
Control group (C)	2–3 years (4 dogs) 4–5 years (2 dogs)	Icelandic sheepdog; Rottweiler; Belgian Tervuren; mixed breed
Study group S1	2–3 years (1 dogs) 4–5 years (2 dogs) 8–9 years (3 dogs)	French bulldog; Rottweiler; mixed-breed
Study group S2	2–3 years (1 dogs) 4–5 years (2 dogs) 6–7 years (2 dogs)	Australian shepherd; Border collie; Rottweiler; mixed breed
Study group S3	6–7 years (2 dogs) 8–9 years (1 dogs) 14 years (2 dogs)	Mixed-breed

**Table 4:** Salivary oxidative stress biomarkers (mean ± standard deviation).

Groups	TAC (U/mL)	SOD (U/mL)	MDA (nmoles/mL)	8-OHdG (ng/mL)
Control group (C)	10.253 ± 1.138	8.515 ± 1.024	14.629 ± 0.443	2.418 ± 0.696
Study group S1	14.942 ± 0.949	13.307 ± 0.644	21.268 ± 1.120	8.233 ± 0.481
Study group S2	15.239 ± 2.936	13.845 ± 2.251	24.444 ± 1.243	11.541 ± 0.644
Study group S3	17.970 ± 0.216	16.854 ± 1.047	27.951 ± 0.528	21.024 ± 1.043

## Data Availability

Data are contained within this article.
